# “Stay Calm and Pick Up Signs to Understand the Cause”: Registered Nurses Use Multiple Strategies for Handling Difficult Emergency Calls

**DOI:** 10.1155/jonm/8964527

**Published:** 2026-09-29

**Authors:** Inger K. Holmström, Hans Blomberg, Ulrika Winblad, Douglas Spangler

**Affiliations:** ^1^ Department of Health Sciences, Innovation and Design, Mälardalen University, Västerås, Sweden, mdh.se; ^2^ Department of Public Health and Caring Sciences, Uppsala University, Uppsala, Sweden, uu.se; ^3^ Uppsala Center for Prehospital Research, Department of Surgical Sciences, Uppsala University, Uppsala, Sweden, uu.se

**Keywords:** authentic calls, difficult calls, emergency medical dispatch centers, telephone nursing, telephone triage

## Abstract

**Background:**

Telephone triage at emergency medical dispatch centers (EMDCs) is often challenging for registered nurses (RNs), and some calls may be difficult to handle. To enable education and interventions for call‐taking RNs, lessons can be learned by studying authentic calls.

**Aim:**

The aim of this study was to describe the strategies used by RNs in difficult calls to EMDCs.

**Methods:**

Call data from 2022 to 2023 were extracted for 14 RNs at three EMDCs in Sweden. Difficult calls were identified based on prior research and operationalized primarily using keyword searches in the free text call notes. During the study period, the 14 RNs handled 27,805 calls. Of these calls, 4888 (17.6%) were identified as potentially difficult and RNs needed to apply different strategies to handle them. A purposive selection of 123 calls was extracted, anonymized, and a deductive content analysis was performed.

**Results:**

The present RNs used eight strategies to handle difficult calls. The most used strategies among the 123 calls were “*pick up signs to understand the cause of the caller’s problem”* (used in 76% of calls) and *“stay calm and focused”* (55%). It was common that RNs applied several strategies during one call, especially if the call was about psychiatric illnesses, including expressions of suicidal intent and calls with vague descriptions of symptoms. Single strategies were used in 20% of calls, combinations of two strategies were used in 50% of calls, while three or more strategies were used in 29% of calls.

**Conclusions:**

RNs typically used combinations of several strategies in handling difficult calls. It is, however, unknown if these strategies were the most appropriate and how they were experienced by callers.

**Implications for Nursing Management:**

RNs need continuous professional development, support from nurse managers and their organization, and further improvements in technical aids to support their handling of difficult calls.

## 1. Background

All registered nurse (RN)–patient communication requires a careful balance of medical‐, nursing‐, and task‐oriented‐ and affective aspects to effectively deliver person‐centered care and promote health [1]. The communication needs to be tailored to achieve optimal health outcomes for patients [2]. Sundler et al. [3] have proposed a framework for person‐centered communication, consisting of five elements: *empathic listening and asking questions, responding and acknowledging, guiding and using clear communication, supportive communication, and appraising* and *confirming communication*. Person‐centered communication thus requires a broad repertoire of skills to be applied and tailored to individuals in various clinical situations and practices. This holds true also for the communication in emergency calls at the emergency medical dispatch centers (EMDCs). Compared to calls to telephone nursing advise services, these calls generally have a higher degree of urgency, including immediate threats to a patient’s life. RNs or dispatchers at EMDCs therefore need a high degree of professional competence and effective communication skills [1] as well as an appropriate clinical decision support system (CDSS) [4, 5]. The challenges include a lack of visual cues, limited or no knowledge about the caller, a high likelihood of acute healthcare conditions, and time pressure [6–8]. According to Schmidt Fotland et al. [9], there are many factors affecting the structure, content, and flow of the conversation: the organization, such as working conditions and CDSS; the RNs’ or dispatchers’ competence, experience, and communication strategies; the caller’s individual characteristics; and the medical problems they present with. The final factor is the interaction between RN and caller, which includes faceless encounters and calls from third‐party callers. For callers to emergency services, crucial aspects include being taken seriously and listened to, as well as being treated empathically [10]. Another important aspect is affirming that callers made the right choice to call the EMDC [10]. However, giving sufficient time to fulfill this during triage [11] can be challenging in busy emergency care settings [9].

Some EMDC calls are more difficult than others. For example, calls about unclear problems [12], calls with communication barriers [8], calls about psychiatric problems [13], and calls from frequent callers were perceived as difficult to handle [14]. In a previous study, 17.6% of all calls were identified as difficult in a call database consisting of 27,805 calls [15], which shows the magnitude of difficult calls and triage decisions encountered by RNs at EMDCs. These calls can put a strain on RNs and create stressful situations, affecting the RNs’ work environment and medical decisions negatively [8]. Difficult calls are likely to have an increased risk of communication errors, which can compromise patient safety and the caller’s experience and satisfaction with care. Handling difficult calls thus requires effective organizational and communication strategies among RNs as well as management support. In an interview study, RNs described strategies used to handle difficult calls: They could use their own professional and personal resources, such as staying calm and focused; use resources within the organization, such as parking the call and handing over to a colleague; or use external resources like taking to someone else at the scene [5]. There may however be aspects of communication patterns and mechanisms that may not be recalled in interviews, which instead can be captured by listening to audio‐recordings of calls [16, 17]. Although studies of authentic EMDC calls exist, they are often focused on specific symptoms such as of subarachnoid hemorrhage [18] or certain populations like pediatric patients [19].

The strategies used by RNs to handle difficult calls in clinical practice are, however, sparsely researched and warrant further study. Most studies in this area are based on self‐reported data, and studies of actual calls are rare but necessary to enable education and interventions. The aim of this study was therefore to describe the strategies used by RNs in difficult calls to EMDCs by analyzing authentic recorded calls.

## 2. Methods

### 2.1. Design

A descriptive observational study with a qualitative deductive approach, based on a constructivist paradigm, was conducted. The constructivist paradigm takes the stance that humans create their own knowledge and understanding of the world through our experiences, social interactions, and language. The data source was authentic audio‐recorded EMDC calls. Such data allow for repeated reviewing to observe and analyze communication behaviors in depth [20].

### 2.2. Sample and Setting

This study was conducted in 3 out of 21 regions in Sweden, which has about 10.5 million inhabitants, and a total of 1.2 million calls per year are made to Swedish EMDCs [21].

In these three regions in mid‐Sweden, EMDCs have all calls handled by RNs, while most of the country’s regional EMDCs relies on dispatchers with varied education and professional backgrounds [22]. RNs are employed at these three EMDCs because their higher competence in nursing, medicine, and communication is considered to provide the best possible quality and patient safety. Together, these three EMDCs employ about 75 RNs and serve about 950,000 people. The EMDCs operate around the clock, all days of the year and handle around 114,000 calls yearly. The calls are routinely audio‐recorded for quality assurance, follow‐up, and clinical education. These EMDCs use a CDSS designed for use by RNs to assess and prioritize calls [23]. The CDSS is developed in‐house and labeled the Medical Decision Support Tool (in Swedish *Medicinskt beslutsstöd*). It is rule‐based and allows the RNs to steer the conversation to suit the situation at hand. The CDSS is structured according to the AMLS assessment mnemonics commonly employed in prehospital care (ABCDE/OPQRST/AMPLE) and follows a layout whereby initial questions focus on ruling out life‐threatening conditions, followed by more detailed, symptom‐specific questions to collect details about the patient’s condition and medical history [23]. RNs are allowed to overrule the CDSS in some situations when it is too urgent and stressful to complete the CDSS to its full extent. In some situations, this is reasonable but might hamper patient safety in other situations [24].

### 2.3. Data Collection

The RNs at the EMDCs were invited to the study via e‐mails and through verbal information during work‐place meetings by the first author, who is an RN with clinical experience from telephone triage. One reminder about the study was sent out in each region’s weekly employee newsletter, as well as two e‐mail reminders to each individual employee. The collected information consisted of a questionnaire with demographic questions and an approval to review their calls for the study purposes. Data including audio‐files as well as patient and call characteristics were extracted from the dispatch system database for the years 2022 and 2023 for all RNs providing informed consent. A total of 14 RNs consented to inclusion in the study. They had a mean age of 45 years (range 27–63 years) and had worked as RNs an average of 15.5 years (range 3–30 years). Five were males and nine were females, and all were native‐Swedish speakers. Out of the 14 RNs, 13 had one or more specialist‐educations, predominantly in prehospital and emergency care. They handled a total of 27,805 calls over the 2‐year study period. Data including telephone number, name, social security number, and address of the caller, as well as the name of the RN handling the call were masked for researchers to assure confidentiality.

Categories of difficult calls were defined based on a previous study, including *calls with communication barriers, calls from agitated or rude callers, calls about psychiatric illness, calls from third parties, calls about rare or unclear situations, calls with unknown addresses and calls regarding immediate life-threatening conditions* [8]. These categories were operationalized using keywords identified in free‐text notes in the CDSS, the volume of repeated patient calls, patient age, and/or dispatch priority, the exact definition of which is described in detail in a previous published study [15], which characterized the difficult calls. Of the 27,805 total calls, 4888 (17.6%) were identified as potentially difficult based on these criteria. As this volume was greater than that which could be analyzed qualitatively, metadata regarding all 4888 calls were then extracted from the dispatch system database, de‐identified, and reviewed by one of the authors who conducted a secondary selection process to determine which calls would be included in the qualitative analysis. Call metadata—including patient demographics, date and time, call duration, number of previous calls, RN and study site, difficult call category, and free‐text notes recorded by the RN—were reviewed to inform purposive sampling. The selection process aimed to maximize variation in patient age and gender; achieve an even distribution across difficult call categories, study sites, and RNs (when possible, including examples from multiple RNs within each category and vice versa); and select calls that were highly representative of the intended meaning of each category. Following the application of the difficult call category operationalization which served as the inclusion criteria, no explicit exclusion criteria were applied. This resulted in a total of 123 calls included in the qualitative analysis, with a median of 9.5 calls per RN (range: 4–14 calls). The audio of these 123 calls was manually de‐identified by an employee of the dispatch centers prior to being delivered to the research group and linked to the call metadata using a sequential identifier associated with each call. A detailed description of the selection process may be found in a paper that describes and characterizes the difficult calls based on the same sample [15].

### 2.4. Analysis

A deductive qualitative content analysis [25] was applied. This approach allows data to be analyzed based on predefined categories derived from research or literature. The researchers use an established set of categories as a guide for coding and interpreting the data to expand existing knowledge while remaining open to new insights that may emerge during the analysis. Data were coded directly from audio‐files, and no transcriptions were made. The analysis was carried out in three phases as described by Elo and Kyngäs [25]: preparation, organizing, and reporting. The preparation phase began with the first author listening repeatedly to the recordings to gain an overview and a general understanding of the data.

The organizing phase included developing a structured analysis matrix derived from Holmström et al.’s [5] study of strategies to handle difficult EMDC calls, as displayed in Table 1. The strategy “*follow the CDSS*” was excluded in the present analysis, as we could not detect to what extent the RNs followed the CDSS from the audio‐recordings. The data were coded by the first author. One of the co‐authors also assessed 20 calls to enhance credibility. The coding was critically discussed among all authors against the matrix. The iterative analysis involved all authors revisiting the steps repeatedly to validate, revise, and refine the findings [26]. Please see Table 1 for a brief description of the matrix and the strategies.

**TABLE 1:** Description of analysis‐matrix with strategies.

Strategies used by EMDC RNs	Brief description of strategy	Example
Pick up signs to understand the cause of the caller’s problem	Listen carefully, picking up verbal and other signs of the caller’s condition. Understanding the causes of potential language barriers (*aphasia?; influenced by drugs?; non-native speaker?)* to handle the problem properly.	RNs listened to signs like breathing and panting.
Provide extra care and time	Provide empathy and supportive nursing care, to reassure and/or distract the caller in distress.	RNs tried to speak gently and be careful with words. They did not want to hang up until caller had calmed down and the situation was under control.
Use simple language and change wording	For non‐native Swedish speakers or very upset callers, the RNs tried to use a simpler language.	RNs exchanged words such as “abdomen” for “tummy” or repeated a question with different wording. Sometimes English was used instead, to see if that eased the communication.
Keep calm and focused	Deliberately stay calm and focused, to instill the feeling of being supported by the RN, and to create co‐operation.	The RNs strove to speak concretely and straight‐forwardly. This meant posing concrete questions and keeping the dialog focused.
Change tone of voice	The change of voice tone could be either to speak louder or to lower the voice to calm down the communication.	By changing the tone of voice, the RNs tried get the caller’s attention and get them to answer questions and follow instructions.
Park the call and hand over	If trying to establish a constructive dialog was not working, the RN could unplug, park the call and ask a colleague for advice or to take over the call.	RNs could tell the caller that “I think it’s better if you talk to a colleague” and hand over the call to achieve a constructive conversation.
Talk to someone else at the scene	If the speech of the caller was non‐comprehensible due to yelling, distress or affected speech, talking to someone else at the scene was a solution.	Asking to speak to a family member, a friend, or someone passing by who might make themselves understood in a better way, and could explain more comprehensively what was happening.
Refer or divert	If the symptoms and situation was non‐urgent and an ambulance was not needed, the caller could be referred to other services.	RNs could for instance refer the caller to a psychiatric clinic, the national telephone nursing line, or other on‐call helplines.
Find a reference location and collaborate with other resources	If the caller was lost and did not know where they were, finding a reference location to start the search for the right address was a common strategy.	The RNs could ask what the point of departure was and then try to start from there to narrow the search field. They could also ask for landmarks like big buildings.
Use interpreters	Using professional interpreters or talk to someone at the scene who spoke Swedish.	The RNs could call an interpreter service, and together with the interpreter make a joint call to the patient.

Throughout the analysis process, recordings were paused for discussions among the authors on communication nuances, and strategies used in the interactions between callers and RNs. This approach is particularly important in communication research, as meaning is conveyed not only through spoken words but also through intonation and context. Finally, the findings are described in the Results section, illustrated with examples of different strategies used in EMDC calls. While a sequential identifier denoting each nurse was available in the call metadata, the ethics approval for the study limited the reporting of results to findings at the aggregate level, precluding the reporting of individual‐level findings. No Artificial Generated Content (AIGC) tools were used for data collection or analysis in this project.

### 2.5. Reflexivity

The research team comprised three senior researchers, two females and one male, and one male post‐doc researcher. Two members of the team were actively working in the EMDC‐context under study, one as consultant and the other as a clinical developer. This involvement may have potentially affected the RNs willingness to participate in the study. However, the participants were informed that their identities would be concealed from the researchers, which may have mitigated this potential influence.

The first author has a background as an RN in nonemergency telephone triage and a longstanding record of publications within this area. The fourth author has a nonclinical background, researching health policy, health care organizations, and health care quality. The study was initiated based on the literature within the field but also based on the authors’ clinical observations and experiences.

The authors’ clinical experiences were discussed in open dialog with the one author with nonclinical experience, to maintain reflexivity.

### 2.6. Ethical Considerations

The study was approved by the Swedish Ethical Review Authority (Dnr. 2021‐06496‐01) and followed the Declaration of Helsinki (2024). RNs were informed that their participation was voluntary, and that they could abstain from participating in any step of the project without providing a reason. While two e‐mail reminders to individual RNs might create a feeling of obligation to participate, this approach was approved by the Ethical Review Authority. The callers were not informed about the study per Ethical Review Authority approval, as all personal information, including names, addresses, and social security numbers, was masked for researchers, and the caller’s approval was hence not needed. The COPE guidelines (https://publicationethics.org/) were adhered to.

## 3. Results

The 123 included difficult calls had a median call duration of 5.6 min, caller being female in 49% of the cases with an average age of 49 years. In 39.5% of the 123 calls, an ambulance with lights and sirens was dispatched, while 33% were sent an ambulance without lights and sirens and 27.5% were referred to other healthcare services or transportation. A more in‐depth quantitative population description may be found in Reference [15]. The distribution of strategies among the 123 analyzed calls and example quotes for each strategy are provided in Table 2. Multiple strategies were often used within one call.

**TABLE 2:** Distribution and examples of strategies applied by RNs in 123 difficult EMDC calls.

Strategies applied in 123 EMDC calls	Number of calls in which the strategy was applied	Percentage of calls in which strategy was applied	Example of RNs’ strategies
Pick up signs to understand the cause of the caller’s problem	93	76%	• *Could you please tell me more?* • *Did this happen before he fainted?* • *Have you been self-medicating in any way?* • *Is that you coughing or is it someone else?* • *Has this happened before? I mean that you’ve been out of breath?* • *Can you please put the phone close to his mouth so I can listen to his breathing?*

Provide extra care and time	9	7.5%	• *You are doing great. Now, keep on with the heart compressions. Help is underway as we speak, and I’ll be here to assist you until the ambulance has arrived.* • *I think that you should not be alone feeling like that, and I don’t want to hang up until you have settled. Now, is there someone you could call to come and stay with you?* • *You seem calm and collected, which is very good indeed. Otherwise, the stress might affect the child as well. Now, I understand this situation is taking its toll on you as a mum. You must be terribly worried…* • *I’m here to help. Now, drop the knife and stop searching for an artery. Go outside to wait for us instead.*

Use simple language and change wording	7	5.5%	• *By pelvis area I mean the hip and groin.* • *Does it hurt? Pain, is she in pain right now?* • *Medication – that’s like pills or drugs. Do you take any pills?*

Keep calm and focused	68	55%	• *Have you done CPR before? Now, lay the child on the table, and I will talk you through it…* • *Please stop yelling – I’m here to help you but we need to establish what has happened.* • *Okey, so he’s hanging there. You need to get him down for me and start CPR.*

Change tone of voice	14	11.5%	• *Yes, I know that you are paying tax and that you want an ambulance, but it is my job to prioritize the ambulances. All are occupied at this moment. I will send one to you but not with lights and sirens…* (voice higher in pitch) • *Hush – please listen to me* (lower tone of voice) *I’m here to help but you need to answer these questions first.*

Park the call and hand over	0	0	—

Talk to someone else at the scene	28	23%	• *Is there someone else with you? Does he or she speak Swedish? Can you hand over the phone to her?* • *Please, talk one at a time! Turn off the loudspeaker and let me speak to your mother.*

Refer or divert	25	20%	• *I understand that your situation is hard and complex, but it’s nothing we can solve right now by sending an ambulance. This can wait until morning, as it is not time-critical and then you can turn to your GP’s office tomorrow.* • *This sounds like an awful situation, and I understand that you need to get your sister out, but I think it’s more of a police matter than a matter of ambulance.*

Find a reference location and collaborate with other resources	25	20%	• *Where did you start this trip? How far from the car are you now? Can you see any buildings nearby, like a church or tower or something?* • *Can you see the lights or hear the sirens from the rescue services vehicles? They should not be far away now, if your position is correct.*

Use interpreters	0	0	—

In the 123 calls analyzed, RNs often used multiple strategies within a single call, as described in Table 3.

**TABLE 3:** Number of strategies used in one call.

Number of strategies used in one call	Number of calls (percent)
One strategy	25 calls (20%)
Two strategies	62 calls (50%)
Three strategies	25 calls (20%)
Four strategies	11 calls (9%)

The most common single strategy in the 123 calls was *“pick up signs to understand the cause of the caller’s problem”.* The most common combination of two strategies was *“pick up signs to understand the cause of the caller’s problem”* and “*stay calm and focused”,* which was used in 16 calls.

When multiple strategies were applied, the calls often concerned psychiatric illnesses including suicidal intentions or calls with an unclear description of the symptoms. *“Extra care and time”* were provided in calls regarding psychiatric illness including suicidal intentions or completed suicide, in addition to calls about immediate life threats to children. When the strategy *“find a reference location and collaborate with other resources”* was applied, the situation was outdoors like a traffic accident, and collaboration was established with the police and/or rescue services. Interpreters were not used in any call, and if language issues arose, the RNs often tried to talk to someone else at the scene. The strategy “*park the call and hand over”* was not used in any of the 123 calls.

When the strategy “*change tone of voice”* was used, the RNs’ voice most often turned higher in pitch and more irritated as the call progressed. In these cases, the caller was often either under the influence of drugs and/or offensive towards the RN. It was apparent that some RNs used a combination of strategies frequently, while others applied them more sparsely. Some of the RNs, for instance, often provided extra care and time, while others focused on ruling out the necessity of a lights and sirens response. As the RNs identities were masked, studying personal communication styles was however not possible in the present data. Some longer excerpts are provided below to further illustrate the strategies used by RNs. Details have been changed to maintain confidentiality. The strategies applied are in italics below.

In the below excerpt, the RNs tries to “*pick up signs to understand the cause of the caller’s problem”,* when a man calls about his partner who has a hernia but now is dizzy and cannot stand by himself. The RN asks multiple questions to get a grip on what is going on, and the severity of it, but the caller provides a vague description of the situation and talks in a slow, low, and somewhat blurry voice:

Caller: Hi…my partner has a stomach hernia and…uhm, uhm…

RN: Yes…

C:… and now he cannot walk, he’s holding on to the walls to keep up…

RN: *Please tell me more, is he in pain?*

C: …hm, I don’t know really, he doesn’t say…

RN: *Are you with him now?*

C: Yes

RN: Can I please talk to him?

(long pause)

C: No, it’s impossible…

RN: *Why?*

C: He doesn’t seem to understand…

RN: *Please tell me more about what’s going on, have you just woken up or what?*

C:…yes, we’ve slept a little.

RN: *And what have you done the rest of the night, have you been drinking or so?*

C:…(long pause) well, there has been some heavy drinking…

In the next excerpt, the RN use the strategy *“refer or divert”* and *“change tone of voice”* when talking to a person who has been calling multiple times the same day regarding several symptoms. This call ends with the RN hanging up, after she has referred the caller to seek help from staff at the residence where the caller lives. As the call unfolds, the RN’s voice gets higher in pitch, and she speaks with irritation and firmness:

C: You need to come and help me! I feel like I have a heart attack, like someone is standing on my chest, it’s hurting so much…

RN: Yes, yes…I can see you have been talking to my colleagues earlier today…

C: But I didn’t get any help and it’s an emergency!

RN: We’ve sent an ambulance twice and they have checked you and taken your vitals…and they are okay, you know.

C: Vitals – I’m not talking about vitals! I have urine and bowel obstruction, and must come to the hospital right away, don’t you see?

RN: *You need to talk to the staff at your residency about those issues.*

C: They don’t care!

RN: *I won’t send an ambulance again, as your vitals are fine. You need to call the staff…*(voice sounds annoyed, RN hangs up)

In the final excerpt, a young mother traveling by car has called about a baby who has stopped breathing. Her location is hard to find, and the RN tries to “*find a reference location and collaborate with other resources”* in addition to *“providing extra care and time”*.

RN: *From what address are you calling?*

C: Well, I don’t know really…I’m at a parking lot outside X‐town… (crying)

RN: How is she doing right now? Is she breathing now? *Can you cope?*

C: I’m not sure, it’s not good at all… (crying)… she stopped breathing and now I think she’s strange in a way, she’s crying but I think she’s like fading away again…

RN: *We will help you, I assure. You are doing great. But where are you now?*

C: At a commuter parking space outside X‐town…

RN: *Let’s see now, I will send help as soon as I’ve located you…outside X-town, but in what direction were you heading?*

C: Heading home along Y‐street…(crying) but now I’ve stopped the car…

## 4. Discussion

The aim of this study was to describe different strategies used by RNs in difficult calls to EMDCs by analyzing authentic recorded calls. The study is hence an observational confirmation and extension of strategies previously identified in a prior interview study rather than identification of new strategies [5].

A combination of different strategies was often used in the 123 analyzed calls, to handle the difficult calls, as previously described in interviews by Holmström et al. [5]. The most common were “*pick up signs to understand the cause of the caller’s problem”,* followed by *“keep calm and focused”.* Multiple strategies were often applied when the calls were extra complex. These findings resemble findings by Eriksson et al. [27], who showed that in a nonemergency setting, RNs described their main strategy “to be calm and secure in themselves.”

The present RNs handling EMDC calls hence seem to use aspects of person‐centered communication proposed by Sundler et al. [3]. These aspects include empathic listening and asking questions, responding and acknowledging, guiding and using clear communication, supportive communication, and appraising and confirming communication [3], which all are present in the 123 calls. We did, however, not study how the callers experienced the calls and the strategies applied by RNs. Most of the previous research on telephone triage and communication has focused on the professional perspective [28]. Mattison et al. [29], however, showed that in a nonemergency context callers rated the interaction as important for overall satisfaction. Although callers’ satisfaction with calls was generally high in their study, there was room for improvement regarding decisional control [29]. Another study from older callers’ perspective concluded that when the communication with the RN was perceived as good and the caller’s perspective was the focus, callers felt safe and satisfied [30]. The scarcity of studies calls for further research from callers’ perspective within an EMDC context. We further do not know if other, perhaps more efficient strategies exist and could have been applied.

Additionally, it is apparent from the 123 audio‐recordings that the RNs had different communication styles and applied the strategies to varying extents. Leaders and managers shape communication patterns and the environment within their teams and influence care coordination and clinical decision‐making [31, 32], which are essential when handling EMDC calls. A personal person‐centered communication style is associated with improved patient outcomes such as satisfaction, increased adherence to advice and treatment plans, and ultimately improved health [28, 33].

It was also evident that in some of the 123 calls, no strategy seemed to suffice, as in the example above when the RNs ended the call by disconnecting or when a call ended in disagreement. For example, *use simple language and change wording”* was only applied in 5.5% of the 123 calls. As the callers often are in distress, this strategy might potentially be useful in more calls as language issues often arose, and Sweden today has a population with about 20% inhabitants born abroad [34]. Knowing this, it is noteworthy that the strategy *“use interpreters”* was not used at all in the 123 calls. This might be due to the urgency of the calls, and using an interpreter service might be perceived to delay proper care for the afflicted person. Marcus et al. [35] argue that access to telephone interpreters might be a satisfactory option, although video remote interpreters are regarded as more effective in other care settings. Interpreters could reduce misunderstandings and facilitate cross‐cultural care encounters [36], and with the rapid development in digital tools, automatic, AI‐driven interpretation might be a useful resource in the near future. Finally, the strategy *“park the call and hand over”* was not applied in any call, but the reason for not using this strategy remains unknown.

### 4.1. Ways Forward

It is unclear if the RNs applied the strategies as a result of their previous experiences and education, given their varied specialist training and years of clinical practice, or whether they had developed a personal communication style by trial and error.

To introduce RNs who are new in the role, training in effective communication and triage should be provided, and educational interventions may improve the handling of difficult calls [12]. Such training and interventions could be both theoretical communication strategies and assessment of unclear and difficult cases, and in workshops with role‐playing or stimulated recall sessions [37]. RNs can also benefit from personal feedback on their communication styles, provided in a constructive and safe environment by mentors and nurse leaders. Continuous professional development is also necessary for RNs who are not new in their role, similar to the development of CDSSs and other systems. It is important that RNs are open to evidence‐based technological innovations which can aid and enhance practice [38]. Assessment and priority setting in difficult calls could also be aided by video live‐streaming. This is implemented in the studied organizations, but there are no strict guidelines on its use, and it was not applied in any of the analyzed calls. Video live‐streaming has since the time of the study been increasingly used in Swedish EMDC settings, and studies show promising results [39, 40].

Supportive leadership and management are essential in strengthening RNs’ competencies [41]. Further studies of how nurse leaders and managers can aid RNs in handling EMDC calls are warranted, as are studies of interventions to support effective and safe strategies in emergency triage. A further question to study is whether dispatchers in other regions who are not RNs communicate in a similar manner and use the present strategies in the same way as RNs.

Given the prevalence of difficult calls, conscious strategies extending the individual RN level are needed. A Finnish study on call‐center RNs concluded that training in communication skills should be provided to meet individual callers’ needs. They also pointed to the need of a supportive work environment to improve caller–RN interactions [42]. This might hold true also for the present EMDC RNs. It would be naïve to think that person‐centered communication [3] and the application of multiple strategies to handle difficult EMDC calls emerge spontaneously. Instead, sustained organizational conditions are required for RNs to develop them. Nurse managers are well positioned to shape these conditions within their teams, for example, by structuring team meetings to include deliberate person‐centered communication practices, investing in communication training, and modeling empathic engagement during clinical interactions [43, 44]. A caring leadership can create positive consequences for RNs, patients, and health care organizations [45].

### 4.2. Strengths and Limitations

The strength of the study includes the large database of authentic EMDC calls, as research in nursing remains dominated by interview and survey studies with a focus on perceptions rather than studies of directly observed behaviors [3]. These authentic audio‐recordings enhance validity by reducing recall bias, capturing real‐time communication dynamics, and allowing analysis of actual communication. The Swedish setting could be a potential limitation, as organization and staffing of EMDC services differ within and between countries, as do guidelines for call‐handling. Regarding the use of audio‐recordings, there is always a risk that participants may adjust their communication behavior, or their interaction becomes less spontaneous when aware of being analyzed in a study. However, the present RNs are used to being recorded, as all calls to EMDCs are continuously recorded for quality assurance and development and they were not aware of the study setup at the time of the recording. The inability to link calls to individual RNs (due to blinding as per Ethical Review Authority approval) restricts analyses of variation between RNs or associations with experience level. The purposive sampling approach used in this study to maximize variation in the selection of calls for qualitative review entails that the prevalence of strategies identified in this study may be different in the full population of difficult calls. Additionally, the deductive analysis approach may limit the identification of new or emerging strategies.

## 5. Conclusions

The present RNs used eight different strategies for handling difficult calls. The most used strategies in the 123 analyzed calls were “*understand the cause and pick up signs”* and *“stay calm and focused”.* It was common that RNs applied several strategies during one call, especially if the call was about psychiatric illness including suicidal intentions or calls with vague descriptions. It is however unknown if these strategies were efficient for handling difficult calls, and how they were experienced by callers. As emergency triage is demanding and requires high competence, RNs need continuous professional development, support from nurse managers and organizations, and further improved technical aids.

### 5.1. Implications for Nursing Management and Leadership

The findings of this study have implications for nursing management, particularly in strengthening communication practices and patient safety. As multiple strategies were used in the most complex calls, training in handling such calls with a range of strategies needs to be provided. Therefore, continuous professional development is essential for RNs to provide accurate triage decisions and wanting to stay in their profession. Leaders and management have a crucial role in supporting and developing the RNs competence and thriving at work, by providing adequate working conditions, education, and feedback to ensure consistency in the ways RNs handle calls. Managers and leaders can model person‐centered communication, and according to Myllymäki et al. [44], the most important factor behind effective competence development and management is probably constant interaction—between the RNs, and between the RNs and the leaders.

## Author Contributions

The study’s conceptual idea and design was a joint effort by all authors. Inger K. Holmström, Hans Blomberg, and Douglas Spangler contributed to data collection. Data analysis, data interpretation, and drafting of the initial manuscript were done by Inger K. Holmström and Douglas Spangler. Inger K. Holmström finalized the manuscript. Proofreading and conceptualization of the manuscript were conducted by Hans Blomberg, Ulrika Winblad, and Douglas Spangler.

## Funding

No specific funding was granted for the present study.

## Disclosure

All authors reviewed and approved the final version of the manuscript.

## Ethics Statement

The study was approved by the Swedish Ethical Review Authority (Dnr. 2021‐06496‐01).

## Consent

The authors have nothing to report.

## Conflicts of Interest

The authors declare no conflicts of interest.

## Data Availability

The dataset generated and/or analyzed in this study is not publicly available due to the highly sensitive nature of individual health status data as per the decision by the Ethical Review Board.
